# Continuous positive airway pressure treatment in sleep apnea: patient compliance and impact on the right heart

**DOI:** 10.1007/s41105-021-00340-x

**Published:** 2021-07-03

**Authors:** Michał Harańczyk, Małgorzata Konieczyńska, Wojciech Płazak

**Affiliations:** 1 Department of Diagnostic Medicine, John Paul 2nd Hospital, Kraków, Poland; 2 Department of Cardiac and Vascular Diseases, John Paul 2nd Hospital, Jagiellonian University Medical College, Prądnicka Str 80, 31-202 Kraków, Poland

**Keywords:** OSAS, Polysomnography, Echocardiography

## Abstract

Obstructive sleep apnea syndrome (OSAS) is considered to be an important predisposing factor for cardiovascular diseases. The main objective of this study was to investigate the impact of CPAP treatment on cardiac structure and function and to assess patient compliance over a long-term course of CPAP treatment. A total of 50 patients diagnosed with moderate-to-severe OSAS based on overnight study, without relevant concomitant diseases were enrolled. Patient compliance, along with echocardiographic and CPAP parameters, was assessed. The average time to follow-up was 38 ± 4.2 months. An increase in tricuspid annular plane systolic excursion (TAPSE) (22.1 ± 4.3–25.5 ± 4.6 mm, *p* = 0.005) and peak early systolic tricuspid annular velocity (S’) (14 ± 3.2–17.2 ± 5.2 cm/s, *p* = 0.005) after CPAP treatment was noted. In patients without CPAP, no significant change in right ventricular (RV) contractility was found. There were no significant differences regarding right atrial (RA) and RV diameters, as well as tricuspid regurgitant peak gradient (TRPG) in both groups; however, a predisposition to increased RA size along with RV and tricuspid annulus diameters was revealed. The mean duration of nightly CPAP use was 3 ± 2.3 h/night in all-day analysis and 4.7 ± 2.1 h/night on days with device usage. The non-adherence rate was 57%. The use of effective CPAP therapy may lead to increased RV systolic function in patients with OSAS in long-term observation. However, long-term patient compliance is generally poor. Regardless of CPAP therapy, a gradual increase in heart size is observed.

## Introduction

Obstructive sleep apnea syndrome (OSAS) is recognized as the most common type of sleep-breathing disorder caused by repeated congestion of the respiratory tract during sleep. Moderate-to-severe OSAS affects about 10% of the middle-aged population with a similar distribution around the world [1]. It is characterized by repeated and numerous episodes of apnea or hypopnea which are observed by others. The main subjective symptoms reported by patients include daytime sleepiness, frequent awakening at night with a feeling of shortness of breath, nocturia, morning episodes of headache, and feeling of dryness in the mouth after awakening. Symptoms reported by patients are often accompanied by loud snoring. Additionally, OSAS is known to play a role in the occurrence of comorbidities [2, 3]. Obstructive sleep apnea syndrome commonly coexists with other respiratory diseases of civilization such as obesity hypoventilation syndrome (OHS), chronic obstructive pulmonary disease (COPD), and bronchial asthma, as well as obesity, which frequently exists as a component of the metabolic syndrome [4, 5]. According to the guidelines, implementation of a continuous positive airway pressure (CPAP) therapy is the recommended first-line treatment for severe OSAS and mild-to-moderate symptomatic patients [6, 7].

The right ventricle (RV) plays an important role in circulatory physiology and pathology. Currently, it is gaining increased interest given its recognized position in many cardiovascular conditions such as heart failure, pulmonary hypertension, coronary artery disease, and arrhythmias [8, 9]. It is known that OSAS may also be related to changes in the RV, resulting in RV remodeling and dysfunction [10, 11]. The main pathomechanism is based on repeated oxygen desaturations during sleep. This causes hypercapnia and acidosis, which may lead to pulmonary vasoconstriction and is finally manifested as pulmonary hypertension. Because echocardiography is the leading evaluation method for increased right-sided pressures, our study focuses on the evaluation of changes in right heart structures using this technique.

Our study was conducted to assess patient compliance to the recommended CPAP treatment and reasons for discontinuing the treatment, to assess the impact of long-term CPAP treatment in patients with moderate-to-severe OSAS on right heart structure and function as evaluated by echocardiography, and to compare RV structure and function in OSAS patients treated or not treated with CPAP during a 3-year follow-up.

## Materials and methods

Our study involved 77 consecutive patients admitted to the hospital due to suspected OSAS.

Eligible subjects more than 18 years of age underwent clinical analysis, including assessment of anthropometric and clinical parameters to eliminate diseases potentially interfering with the study. The Epworth questionnaire (ESS), regarding symptoms of OSAS, daytime sleepiness, and tiredness, was completed by study subjects.

The exclusion criteria were as follows: inability to perform the testing procedures or to self-operate CPAP device, heart failure of any etiology, diminished ejection fraction (< 50%), congenital heart disease, severe or moderate valvular disease, uncontrolled arterial hypertension, history of pulmonary embolism, uncontrolled or severe asthma, chronic obstructive pulmonary disease (COPD) or any other pulmonary disease, pulmonary hypertension, other untreated or uncontrolled diseases (diabetes mellitus, hypo/hyperthyroidism, renal failure, hepatic failure), patients treated previously with CPAP or any other effective treatment for OSAS.

We performed an overnight recording using a sleep-monitoring system (Embletta MPR, type III according to the American Academy of Sleep Medicine, AASM) in all subjects [12]. Standard criteria of sleep event scoring were used. Obstructive apnea (OA) was scored when there was an absence or reduction to less than 10% of the baseline airflow with continued respiratory effort, lasting 10 s or longer. Central apnea (CA) was scored as an absence or reduction to less than 10% of baseline airflow without continued respiratory effort, lasting 10 s or longer. Hypopnea (H) was scored as a decrease in airflow of at least 30% of baseline for 10 s or more accompanied by a 3% or more decrease in oxygen saturation. When an event met apnea criteria and was associated with absent inspiratory effort in the initial part of the event, followed by resumption of inspiratory effort in the second part of the event, it was scored as a mixed apnea (MIX). The AHI was expressed as the sum of the number of apneas plus the number of hypopneas that occurred, on average, each hour.

According to sleep study, 50 patients were diagnosed with moderate-to-severe OSAS with an apnea–hypopnea index (AHI) ≥ 15/hour. All subjects gave their informed consent before participating in the study.

### CPAP adjusting

All patients diagnosed with moderate-to-severe OSAS were recommended to make behavioral changes such as discontinuation of smoking and alcohol intake, as well as reducing body mass, if their BMI exceeded 25. Prior to initiation of treatment, a mask-fitting test was performed for each patient. To minimize large leaks during titration, only oronasal masks were used. The in-hospital titration protocol included two consecutive nights during which effective therapeutic pressures were adjusted. Initial pressure settings were usually 7–13 cmH_2_O and expiratory pressure relief was set to 3 cmH_2_O. However, during the titration process, various CPAP device settings were used, with adjustments performed according to the obtained parameters. In all cases, we enabled the “ramp” setting, which initiates airflow at a low level and slowly increases the pressure to the set level in order to make titration more comfortable for the patient. Two types of devices were used: RESMED AutoSet S9 (ResMed, Bella Vista, Australia) and REMstar Auto (Philips-Respironics, Murraysville, PA, USA). No humidifiers were used during the titration protocol. Initial therapeutic pressure level, respiratory relief level, and therapeutic pressure rise time were adjusted individually based on a patient’s comfort or clinical response.

### Echocardiography

A day before CPAP titration and at the follow-up visit, we performed two-dimensional (2D) echocardiographic imaging in standard views, including conventional 2D, Doppler, Color Doppler, and Tissue Doppler images (TDI), using a Philips IE33 device (transducer X5-1; 1.3–4.2 MHz) [13]. The examination was performed in left-lateral decubitus and supine positions. For right heart assessment, the parasternal long axis (PLAX) and RV-focused 4-chamber view was used. Left PLAX view was used for the measurement of the proximal portion of the right ventricular outflow tract (RVOT). Additionally, basal RV (RVD1), as well as mid-cavity RV (RVD2) and RV longitudinal (RVD3) dimensions were acquired. The tricuspid regurgitant jet was imaged from different views and the maximal velocity was recorded. The pulmonary artery pressure was calculated using the modified Bernoulli equation [14]. The M-mode technique was used to assess the maximum systolic excursion of the lateral tricuspid annulus (TASPE) in an apical 4-chamber view. Tissue Doppler imaging (TDI) was performed to obtain peak systolic (S’) velocity and peak early (E’) diastolic myocardial annular velocity. Once the optimal 2D image was obtained, acquisition was made in the form of five heart-beats, enabling us to choose the most reliable measurements of dimensions and Doppler wave forms.

### Statistical analysis

Continuous variables are presented as mean and standard deviations (SD). The Shapiro–Wilk test was used to determine normality of variable distribution. The paired *t* test for continuous variables was conducted to assess for differences between the groups. The accepted statistical significance threshold was established as a *p* value of < 0.05.

## Results

### Patient characteristics

A total of 50 consecutive patients were included in the final analysis (Table 1). Out of these 50 patients, 9 (18%) individuals did not meet CPAP qualification criteria, i.e., the AHI was below 30/h and they were not characterized by daytime sleepiness or any other relevant concomitant disorders requiring CPAP treatment. One patient (2%) refused CPAP treatment for personal reasons, one patient was referred to the pulmonology department in order to confirm coexisting OHS, and one patient had a contraindication to CPAP therapy (chronic untreated sinusitis). From patients allocated to conservative treatment, 7 (14%) patients were lost to follow-up. The remaining 38 (76%) individuals were trained in the use of the CPAP device and appropriate equipment for therapy was selected. The average time from enrollment to follow-up visit was 1138.8 ± 125 days (38 ± 4.2 months).

**Table 1:** Clinical characteristics of OSAS patients at the follow-up

		CPAP ( +)	CPAP ( −)
		*n* = 16	*n* = 22
Male/female	n/n	12/4	12/10
Age	(years)	60.1 ± 9	63.4 ± 10.8
BMI		35 ± 5.1	32.7 ± 5.1
Hypertension	*n* (%)	15(94%)	17(77%)
DM type 2	*n* (%)	5(31%)	6(27%)
Prediabetes	*n* (%)	5(31%)	6(27%)
Hypercholesterolemia	*n* (%)	14(88%)	21(95%)
Active smokers	*n* (%)	6(38%)	4(18%)
Ex-smokers	*n* (%)	4(25%)	5(23%)
Beta-blockers	*n* (%)	8(50%)	12(54%)
Statins	*n* (%)	8(50%)	12(54%)
Diuretics	*n* (%)	6(38%)	7(32%)
LVEF	(%)	62.8 ± 5.6	63.3 ± 3.6
Time to follow-up	(Months)	38 ± 4.1	38 ± 4.3

Data expressed as mean ± SD or number (%) of patients

*OSAS* obstructive sleep apnea, *CPAP* continuous positive airway pressure, *SD* standard deviation, *BMI* body mass index, *DM type 2* diabetes mellitus type 2, *LVEF* left ventricular ejection fraction

### CPAP treatment

**Fig. 1:**
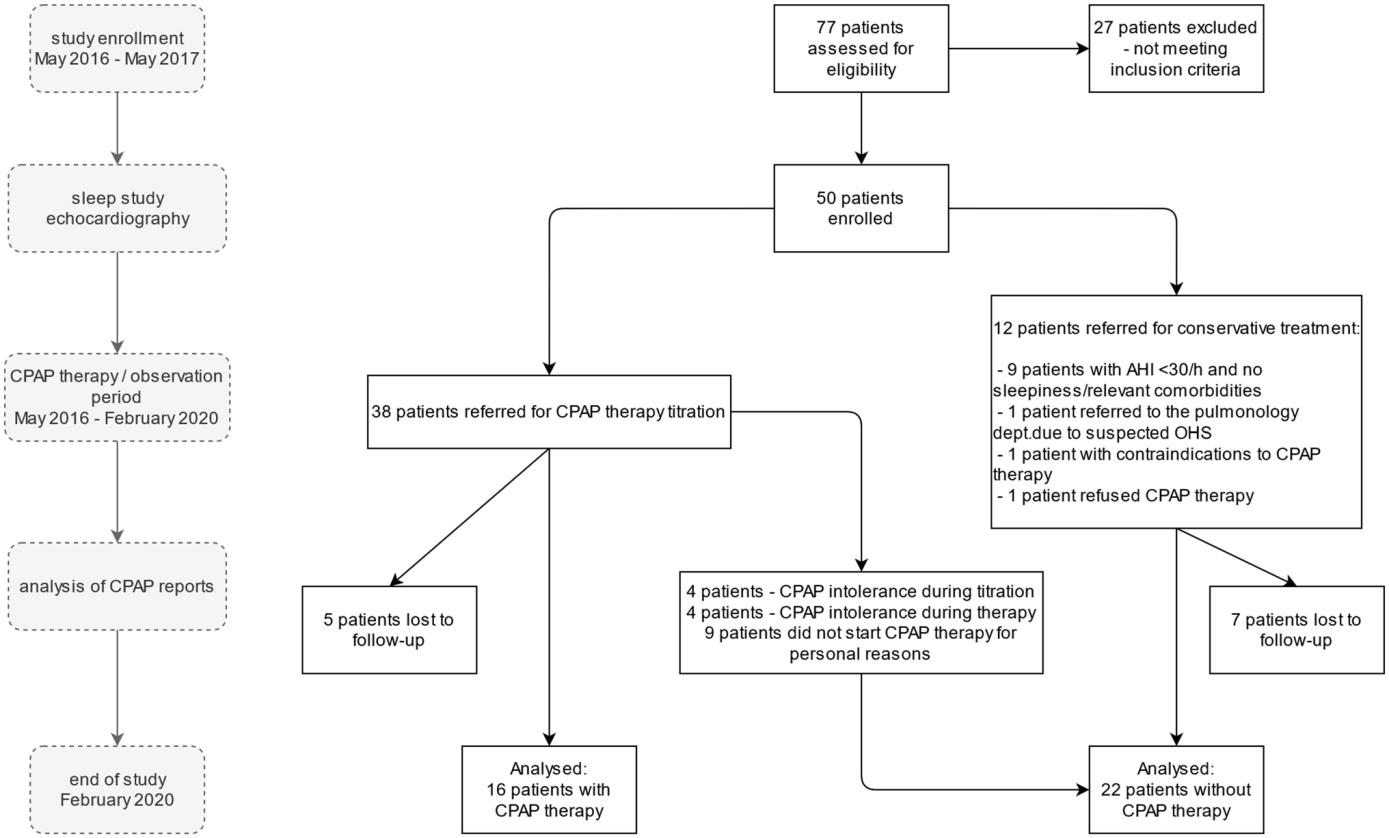
Flow-chart identifying patients enrolled in the study and available for analysis

Out of 38 patients available for analysis, CPAP was not recommended in four patients due to the presence of moderate OSAS and the absence of significant clinical symptoms, mainly manifested as low daytime sleepiness and absence of significant comorbidities. One patient had a contraindication for CPAP therapy, as mentioned above. Nine patients refused the proposed CPAP therapy, did not report for titration for their own reasons, or did not initiate therapy after titration. Due to poor tolerance during CPAP titration, four patients withdrew from treatment and four patients discontinued using the device after a few months due to poor treatment tolerance during its course. Data regarding CPAP therapy from these patients were not available for further analysis. None of the patients reported discontinuation of CPAP treatment due to its side effects. The final analysis was based on data from16 patients who were treated with CPAP therapy and 22 patients who were not treated or reported poor tolerance to CPAP. This is presented in Fig. 1.

Successful CPAP therapy, defined by a residual AHI < 5/h as measured by the device algorithm, was achieved in 13 (81%) patients. The mean residual AHI was 2.7 ± 2.6/h. Reports on device usage were available for a mean of 17.2 ± 9.5 months and were downloaded from devices during the follow-up visit. The overall CPAP non-adherence rate was based on a 7.0 h/night sleep period, which was recommended in previous studies [15, 16]. The non-adherence rate, calculated by subtracting 3.0 h from 7.0 h, then dividing this by 7.0 h and converting to a percentage, was 57%. The results on CPAP therapy are presented in Table 2.

**Table 2:** Results of CPAP therapy

		CPAP patients at baseline *n* = 16	CPAP patients at follow-up *n* = 16	*p* value
AHI	(h¯^1^)	46.3 ± 18.5	2.7 ± 2.6	< 0.0001
ODI	(h¯^1^)	42.9 ± 21.8	–	–
Sat	(%)	90.1 ± 4	–	–
OA	(h¯^1^)	23.6 ± 22.2	0.5 ± 0.5	< 0.0001
H	(h¯^1^)	15.2 ± 9.5	1.4 ± 1.8	< 0.0001
CA	(h¯^1^)	1.8 ± 3.4	0.1 ± 0.2	< 0.0001
MIX	(h¯^1^)	5.7 ± 6.7	0	< 0.0001
ESS		10.5 ± 6	–	–
Av. CPAP press	(cmH_2_O)	–	10.4 ± 1.7	–
Duration of CPAP usage (all-day analysis)	(h/day)	–	3 ± 2.3	–
Duration of CPAP usage (DwDU)	(h/day)	–	4.7 ± 2.1	–
Weighted mean for CPAP use^†^	(h/day)	–	2.4 ± 2.4	–
%DwDU	(%)	–	53.2 ± 30	–
DwU4H	(days)	–	146.2 ± 127.3	–
%DwU4H	(%)	–	41.2 ± 32.5	–

Data expressed as mean ± SD

*AHI* apnea–hypopnea index, *Av. CPAP press* average CPAP pressure, *CA* central apnea, *CPAP* continuous positive airway pressure, *DwDU* days with device usage, *DwU4H* days with device usage greater than 4 h, *ESS* Epworth Sleepiness Scale score, *H* hypopnea, *MIX* mixed apnea, *OA* obstructive apnea, *ODI* oxygen desaturation index, *Sat.* Saturation, *%DwDU* number of days with device usage divided by number of reported days, *%DwU4H* days with usage ≥ 4 h/night divided by days with device usage

^†^Weighted mean for CPAP use was calculated as mean usage for all 20 patients who started CPAP treatment at baseline

### Echocardiography

An increase in RV systolic function was observed in patients with CPAP treatment, expressed as an improvement in TAPSE and S’ (Table 3). In this group, average TAPSE at baseline (22.1 ± 4.3 mm) significantly increased during the follow-up period (25.5 ± 4.6 mm, *p* = 0.005). Significant improvement in S’ before (14 ± 3.2 mm) and after (17.2 ± 5.2 cm/s, *p* = 0.005) CPAP treatment was also noted. There was no significant difference in TDI E’ for both groups. In the group without CPAP therapy, there was no significant change in RV systolic parameters. Analysis of RA and RV diameters, as well as TRPG, did not reveal significant differences during the time of observation; however, a tendency toward increased dimensions was observed for RA size, RVD1, RVD3, and tricuspid annulus diameters. A similar trend was observed in the tricuspid valvular regurgitation gradient.

**Table 3:** Echocardiographic parameters describing right heart structure and function

		Patients treated with CPAP *n* = 16			Patients without CPAP *n* = 22		
		Baseline	Follow-up	*p* value	Baseline	Follow-up	*p* value
RVOT	(mm)	31.8 ± 4.3	32.9 ± 2.9	ns	32.0 ± 2.5	30.0 ± 7.2	ns
TAPSE	(mm)	22.1 ± 4.3	25.5 ± 4.6	0.005	23.7 ± 5.3	25.8 ± 4.6	ns
RV S’	(cm/s)	14.1 ± 3.2	17.2 ± 5.2	0.005	15.0 ± 3.9	15.1 ± 2.8	ns
RV E’	(cm/s)	11.4 ± 2.8	11.5 ± 2.5	ns	11.6 ± 3.1	10.8 ± 2.9	ns
RAd1	(mm)	46.2 ± 12.1	50.6 ± 7.9	ns	42.9 ± 7.2	43.4 ± 7.1	ns
RAd2	(mm)	49.4 ± 5.4	50.1 ± 9.2	ns	48.6 ± 6.2	49.4 ± 6.6	ns
RAA	(cm2)	20.7 ± 2.7	22.9 ± 6.7	ns	18.6 ± 4.4	19.0 ± 4.7	ns
RVD1	(mm)	44.1 ± 5.2	45.9 ± 6.4	ns	41.4 ± 5.6	42.2 ± 5.5	ns
RVD2	(mm)	36.6 ± 5.7	37.3 ± 6.8	ns	33.6 ± 6.6	32.6 ± 5.2	ns
RVD3	(mm)	74.9 ± 28.0	75.1 ± 22.6	ns	71.3 ± 22.2	73.4 ± 20.8	ns
TRPG	(mmHg)	22.9 ± 6.2	23.6 ± 6.7	ns	24.2 ± 4.6	25.3 ± 6.8	ns

Data expressed as mean ± SD

*CPAP* continuous positive airway pressure, *RVOT* right ventricular outflow tract, *TAPSE* tricuspid annular plane systolic excursion, *RV S* peak early systolic tricuspid annular velocity, *RVE* peak early diastolic tricuspid annular velocity, *RAd1* right atrial transverse dimension, *RAd2* right atrial longitudinal dimension, *RAA* right atrial area, *RVD1* right ventricular basal dimension, *RVD2* right ventricular mid-cavity dimension, *RVD3* right ventricular longitudinal dimension, *TRPG* tricuspid regurgitant peak gradient, *ns* not statistically significant

The subgroup analysis revealed significantly higher RV contractility presented as TAPSE and S’ in patients aged under 60 years, when compared to patients aged 60 years or older, as presented in Table 4. At the follow-up significant increase of TAPSE and S’ in patients aged under 60 years was noted. Furthermore, improvement of RV systolic function was observed in patients with a BMI less than 35 and with a BMI of 35 or higher.

**Table 4:** Subgroup analysis of RV systolic parameters according to age and weight

		Baseline	TAPSE		Baseline	RV S’	
			Follow-up	*p* value		Follow-up	*p* value
Age (years)	< 60	23.2 ± 3.7	27.6 ± 4.6	0.01	15.1 ± 3.1	19.8 ± 5.1	0.01
	≥ 60	20.6 ± 4.8	22.8 ± 3.1	ns	12.7 ± 2.9	14.9 ± 3.3	ns
BMI (kg/m^2^)	< 35	20.7 ± 3.8	23.8 ± 4.8	0.04	13.1 ± 2.5	15.1 ± 4.4	0.04
	≥ 35	24.3 ± 4.6	27.0 ± 4.2	0.02	15.3 ± 3.7	20.1 ± 5.0	0.01

Data expressed as mean ± SD

*BMI* body mass index, *TAPSE* tricuspid annular plane systolic excursion, *RV S’* right ventricle, *RV S’* peak early systolic tricuspid annular velocity, *ns* not statistically significant

## Discussion

Previous studies examined subjects in various stages of OSAS who received CPAP therapy. They observed a significant improvement in daytime sleepiness and fatigue, as well as less frequent nocturia, reduced snoring, and decreased number of awakenings during sleep. They also found that CPAP therapy could restore correct sleep patterns, improve concentration and quality of life, and prevent motor vehicle accidents [17]. However, its impact on cardiovascular events, such as myocardial infarctions or heart failure, remains controversial [18].

### Compliance

To the best of our knowledge, this is the longest observation period of patients undergoing echocardiographic assessment of right heart structures and hemodynamics in patients with OSAS treated with CPAP. A significant proportion of patients either did not initiate or, at some point during the study, discontinued CPAP therapy.

Reasons associated with the decision to initiate or discontinue treatment in our study were consistent with those reported in literature. The most important factors that discouraged treatment initiation among study participants were costs of CPAP machines and accessories, negative experiences with CPAP therapy in relatives, and expected length of treatment—as CPAP should be considered as a lifelong treatment for patients with OSAS. Common reasons for discontinuation of therapy mentioned by patients included: lack of motivation, no remarkable treatment effects, and the experience of nasal congestion or irritation.

The poor compliance observed in our study may have been due to the fact that we used a longer observation period when compared to other studies which evaluated patient compliance. Moreover, it is known that one of the factors affecting compliance is patient awareness of being monitored, which was neutralized in our investigation by the lack of follow-up visits during the study. To reflect the natural course of patient treatment and their susceptibility to treatment, during the follow-up period there were no scheduled periodic visits, patients did not receive any messages reminding them to use the recommended treatment, and no motivational techniques were used which could have affected patient compliance. Patients had the opportunity to use standard medical visits in a local pulmonology ambulatory clinic, during which they were able to have their CPAP therapy assessed. However, this is similar to the general model of CPAP-patient care within the state health insurance system.

In contrast to our study, greater compliance was noted when assessing patients having a shorter treatment period along with a follow-up visit midway through the observation period. A study by Dinh-Thi-Dieu et al. [19] examined patient adherence to auto-CPAP therapy in a short-term follow-up after receiving auto-adjusting CPAP treatment at home. In this group of 139 patients, the number of days with CPAP usage at night was 26.6 ± 1.8 days/month, while only five patients discontinued therapy for various reasons.

Furthermore, previous investigations have reported that early pressure modification enhances CPAP adherence during the first 6 months of therapy [20]. In this study, only 13% patients stopped CPAP therapy, and 17% patients were non-compliant. Similarly, a meta-analysis of CPAP compliance which compared telemonitor care with usual care, indicated that telemonitoring follow-up could significantly increase CPAP compliance [21]. We hypothesize, that using of some influence techniques mentioned above could gain patients’ enhancement. However, a recent systematic review reported that the general non-adherence rate remained high (34.1%), with mean non-weighted duration of nightly use, weighted mean CPAP use, and percentage of use being 4.6 h, 4.46 h and 36.3%, respectively [16]. For individuals with poor compliance or who cannot tolerate CPAP, hypoglossal nerve stimulation or surgical procedures or intraoral devices may constitute useful second-line therapy [22].

### Echocardiography

Our study showed that the use of CPAP results in improved RV systolic function as measured by TAPSE and S’. These findings are in line with the results of a 1-year study by Karamanzanis et al. [23] (CPAP use: 85.2 ± 17% of nights), which also demonstrated an improvement in TAPSE and RVD. Another study evaluating changes in RV systolic function showed a significant improvement in TDI parameters [24]. This group found that a 6-month CPAP treatment period with good compliance (CPAP use: 6.8 h/night) led to structural and functional changes resulting in RVD decrease and S’ increase [24]. Moreover, our results share a number of similarities with Yoshihisa et al. [25] findings, who claims that 6-month positive airway pressure therapy improves right heart and pulmonary function, expressed as right ventricular fractional area change (RV-FAC), tricuspid valve regurgitation pressure gradient (TRPG) and tricuspid valve E/E’ (CPAP or ASV use: 5.9 ± 1.3 h/night). Furthermore, Dursunoglu et al. [26] observed that RV global dysfunction expressed as a high myocardial performance index (MPI) at baseline, was significantly decreased after 6-month CPAP therapy (CPAP use: 5.6 ± 1.7 h/night).

Nevertheless, short-term studies might not show any differences in RV systolic function when assessing TAPSE and S’, as was mentioned in a 4-month observation by Vitarelli et al. [27] and a 6-month study by Hammerstingl et al. [28] (CPAP use: 6 ± 0.3 and 6.5 ± 1.1 h/night, respectively). Similarly, a 3-month comparison of CPAP vs. sham CPAP treatment did not improve S’ in any of the groups, which was also noted in a study of patients receiving 6 months of CPAP treatment by Bayram et al. (CPAP use: 6.4 ± 2.2 h/night) [29, 30]. This can be explained by the short time of treatment to obtain the improvement in RV systolic function. Similarly, there were no significant changes in RV systolic function as assessed by the global myocardial function index in a study evaluating patients with OHS in which CPAP or noninvasive ventilation (NIV) treatment were used for 3 years [31]. In this group of patients, 73% had OHS/OSAS overlap. The median treatment adherence for CPAP was 6.0 h/day; however, patients were encouraged to improve adherence during annual visits.

Current data in the literature concerning the impact of CPAP therapy on cardiovascular events are contradictory. A recent meta-analysis did not show a relevant reduction in significant endpoints (ischemic strokes or coronary events) in the 6 analyzed randomized controlled trials (RCT) [32]. On the contrary, a significant effect of CPAP was demonstrated in the 11 non-RCTs, but these studies were characterized by a higher degree of patient adherence than in RCTs [32]. Similarly, there was no difference in all-cause mortality analysis when only the results of RCTs were taken into account [32]. Other studies suggest that positive airway pressure may improve prognosis in patients with heart failure and sleep-disordered breathing with favorable effects such as improvement of cardiac systolic or diastolic function [33, 34]. In our study, we observed a tendency toward increasing dimensions of right heart structures, which strongly suggests a lack of reverse remodeling and seems to confirm the results of this meta-analysis. It can be assumed that subclinical changes occurring in the right heart, despite adequate CPAP treatment, may result in serious cardiovascular events in the future; however, further research in this field is needed.

### Limitations

A limitation of our study was the presence of a number of inherent clinical confounders such as obesity, hypertension, diabetes, or prediabetes in our group of patients. These confounding factors may influence the natural course of OSAS in an otherwise healthy population. Another limitation of our study was the inability to isolate the impact of aging in the observed population over the study duration, which has an independent effect on the deterioration of right heart parameters. Moreover, the CPAP non-users group in fact was made up of a mixed group of patients: five referred at the outset for conservative treatment and 17 who did not start CPAP for different reasons mentioned in manuscript, even though they met the treatment criteria. As the control group consists of patients who were not adherent during the follow-up period, i.e., a post hoc control group, a selection-bias cannot be excluded. Finally, the relatively low compliance in our patient group constitutes a potential limitation of this study. Nonetheless, it should be noted that CPAP treatment is often a poorly tolerated therapy and problems related to patient compliance are frequently present in most CPAP studies. In conclusion, the use of effective CPAP therapy may lead to an increase in RV systolic function in patients with OSAS during long-term observation. However, long-term patient compliance is generally low. A lack of willingness to follow the recommended treatment along with intolerance during titration or therapy are among the most common causes of treatment refusal. Regardless of CPAP therapy usage, a gradual increase in heart size is observed. Further prospective studies are needed to determine the effect of the observed changes on the clinical outcome of OSAS patients.
